# Serological Predictors of Protection from Infection Upon Household Exposure to SARS‐CoV‐2

**DOI:** 10.1002/eji.70269

**Published:** 2026-08-24

**Authors:** Henrike Maaß, Imke Hinrichs, Martina Pavletic, Manuela Harries, Tatjana Prinke, Najat Bdeir, Richard Egelkamp, Berit Lange, Yannic C. Bartsch, Mate Lerga, Luka Cicin‐Sain

**Affiliations:** ^1^ Department of Viral Immunology Helmholtz Center for Infection Research Braunschweig Germany; ^2^ Centre For Individualised Infection Medicine (CiiM) a joint venture of Helmholtz Centre for Infection Research and Hannover Medical School Hannover Germany; ^3^ Laboratory of Anti‐Viral Antibody‐Omics, TWINCORE‐Institute for Experimental Infection Research Helmholtz Center for Infection Research (HZI) and Medical School Hannover (MHH) Hannover Germany; ^4^ Emergency Department Clinical Hospital Center Rijeka Rijeka Croatia; ^5^ Department for Anesthesiology and Reanimatology Emergency and Intensive Care Medicine, Medical Faculty Rijeka Rijeka Croatia; ^6^ Department of Epidemiology Helmholtz Center for Infection Research Braunschweig Germany; ^7^ Public Health Agency of Lower Saxony (NLGA) Hannover Germany; ^8^ German Centre for Infection Research (DZIF) Partner Site Hannover/Braunschweig Braunschweig Germany

**Keywords:** antibody titers, correlate of protection, COVID‐19, functional assay, neutralizing antibodies, SARS‐CoV‐2, serology, vaccine

## Abstract

Correlates of protection against symptomatic and severe breakthrough SARS‐CoV‐2 infections are well characterized. However, correlates of protection against virus transmission are poorly defined due to a lack of evidence in well‐designed prospective clinical trials. We studied a Croatian cohort of individuals with documented household exposure to SARS‐CoV‐2 in late 2022/early 2023. Sera were acquired at the time of the COVID‐19 diagnosis of the index case and before symptom onset of the test case. Samples were comprehensively analyzed for predictors of protection against virus transmission. PCR‐negative participants at day 0 were recalled 14 days later and re‐tested by PCR and IgM ELISA to identify any virus transmission, including asymptomatic ones. Out of nearly 200 tested serological parameters, several serological features differed between the participants that became PCR‐positive during the study period and those that remained PCR‐negative, although none remained significant after correction for multiple testing in the full cohort. Titers of variant‐specific neutralizing antibody showed the biggest difference and were higher in the uninfected subgroup. Since recent antigenic exposure may confound results, we identified the recipients exhibiting IgM seroconversion and censored them from the study. This refinement clearly separated infected and uninfected individuals by variant‐specific neutralization titers and IgA1 responses to BA4/5 RBD, which remained significant after correction. Therefore, our data indicate that high IgA1 and neutralizing titers may be predictive serum correlates of protection against SARS‐CoV‐2 transmission in intense contacts among household members, but only if variant‐specific antigen is used in serological assays.

## Graphical abstract

Household members of SARS‐CoV‐2 positive index persons donated a serum sample (*t* = 0). Within 14 days, the household members reported if COVID‐19‐like symptoms appeared and returned after 2 weeks to be retested. Serum responses were comprehensively measured and elevated variant‐specific IgA1 and neutralizing antibody responses identified in the noninfected group.

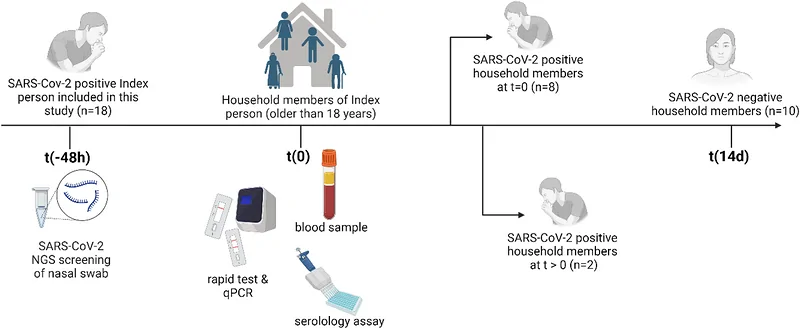

## 1 Introduction

The coronavirus disease that emerged in December of 2019 (COVID‐19), caused by the Severe Acute Respiratory Syndrome Coronavirus 2 (SARS‐CoV‐2), has had a profound impact on public health, leading to widespread infections, disease, and death. The establishment of herd immunity due to the spread of infections and global vaccine campaigns has led to a reduction in severe symptoms. However, infection with SARS‐CoV‐2 can still result in long‐term complications, known as long COVID, regardless of the initial severity of the disease [1], and vulnerable populations, such as the immunocompromised or the elderly, remain at risk of severe disease courses. Anti‐COVID vaccines prevent severe disease, yet their effectiveness against symptomatic infections and milder disease courses wanes relatively quickly [2]. As a result, vaccination recommendations are primarily focused on individuals at higher risk of developing severe COVID‐19, such as the elderly, immunocompromised individuals, and those working with vulnerable populations [3]. Preventing infections in general, not just severe disease, should remain important in light of the risk of long COVID, which is common even upon less severe acute episodes, as it has been shown that vaccination reduces the risk of long COVID as well [4].

Since the development of SARS‐CoV‐2 vaccines, correlates of protection (CoP) have been studied to assess vaccine efficacy. Early research identified neutralizing antibodies (nAbs) as key CoPs against symptomatic disease induced by both vaccination and infection [5, 6, 7]. However, no CoP or threshold levels have been established to guide recommendations on protection against infection, and hence, transmission. Therefore, national advisory bodies provide widely divergent guidelines on booster vaccination schedules. Most studies investigating CoPs have merely provided estimates based on the time since last vaccination, without knowing the exact time or circumstances of participants' exposure to SARS‐CoV‐2 or objective criteria to quantify immune responses [8, 9]. However, there are few studies assessing the role of serological parameters predicting protection against virus transmission, especially in light of the rapidly evolving variants of SARS‐CoV‐2. Recent household studies indicate that protection from infection is neutralizing‐antibody‐mediated in a variant‐specific manner, with titers to the circulating variant most predictive Recent household studies indicate that protection from infection is neutralizing‐antibody‐mediated in a variant‐specific manner, with titers to the circulating variant most predictive [10, 11], and that serological markers reveal substantial undocumented infection [12].

In this study [13], we examined household members where one individual (the index case) tested positive for SARS‐CoV‐2 in the emergency department of the Clinical Hospital Center Rijeka, Croatia, during the winter of 2022/2023. To estimate the protection of transmission within the households, we analyzed serological parameters and neutralizing antibodies. Household members were tested at an initial time point shortly after the index case and again 14 days later, or earlier if they developed COVID‐like symptoms. Based on their SARS‐CoV‐2 status during this period, household members were categorized into SARS2 positive and those that remained SARS2 negative throughout the analysis period and comprehensively analyzed to identify differences in serum parameters between those two groups. Variant‐specific nAbs, along with various other serum features such as variant‐specific IgA1 titers, were the best predictors of protection against virus transmission, while vaccination status and the timing of the last immunogenic event were not significantly predicting the protection against breakthrough infections.

## 2 Results

### 2.1 Demographic and Clinical Characteristics of Study Population

**FIGURE 1:**
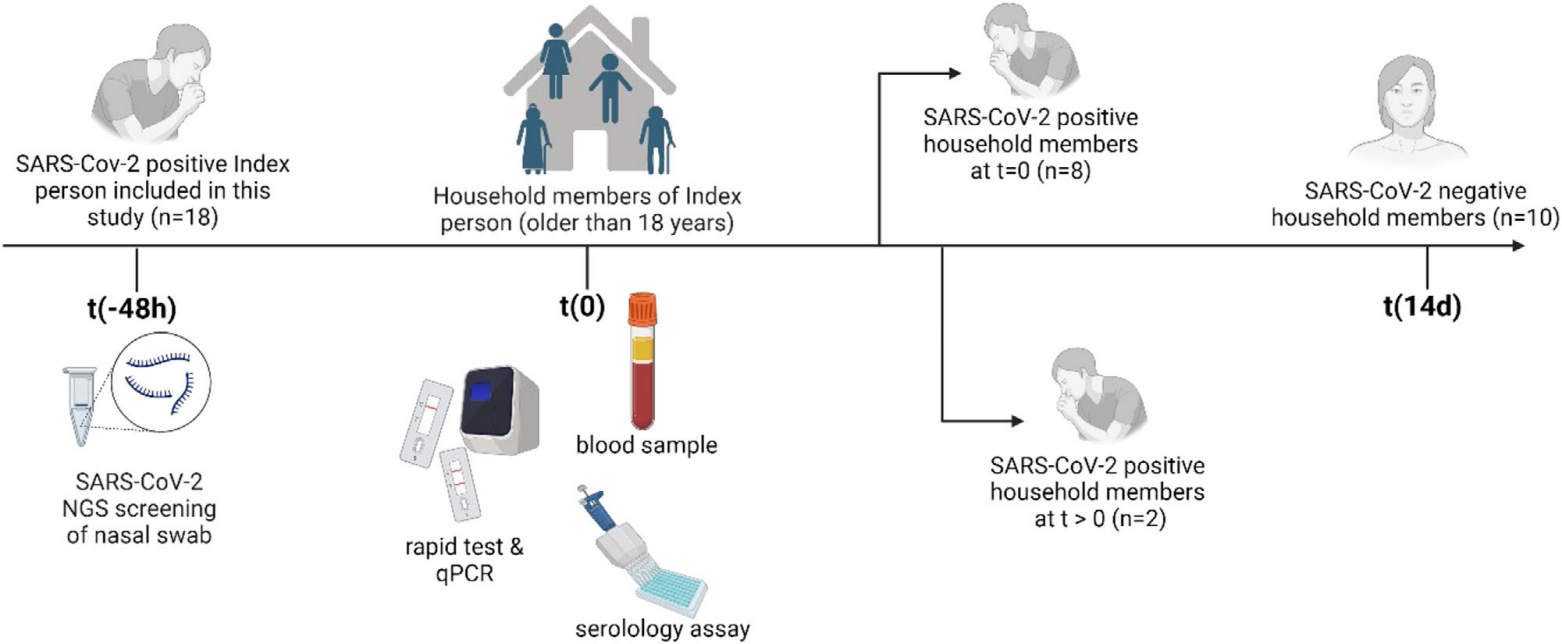
Brief schematic study overview. Household members of SARS‐CoV‐2 positive index persons donated a serum sample (*t* = 0) at the Clinical Hospital Center Rijeka, 48 h after confirmation of the infection of the index person. Within 14 days, the household members were asked to report if COVID‐19‐like symptoms appeared or were asked to come back after 2 weeks to be tested by rapid test and qPCR. Serology features were determined using various assays. Created with BioRender.com.

This study was conducted at the Clinical Hospital Center Rijeka, focusing on households in which one member tested positive for SARS‐CoV‐2 (henceforth called the index person). Household members donated a blood sample within 48 h after the index person tested positive. The samples were analyzed, categorizing the household members based on whether they contracted the infection within 14 days after the initial sampling or remained uninfected. A schematic study overview can be seen in Figure 1 and Figure S1.

The study cohort consisted of 18 index people and 20 household members. RNA from nasal swabs of index cases was sequenced to identify the virus variant and perform variant‐adjusted serology. While Omicron BA.5 was the most common variant in this group, other variants, such as BA.2.75, BF.7, or BQ1, were also present (Figure S2). Half of the household members (*n* = 10) remained negative by PCR over the 14‐day study period (henceforth called not‐infected or negative group). Eight household members were SARS‐CoV‐2 positive at the initial testing, and two were infected within the 14 days (these 10 participants will be referred to as the infected or positive group). Both groups presented similar characteristics, which can be seen in Table 1. In summary, both groups showed no significant differences in terms of number of vaccinations or days since last vaccination or latest immunogenic event (i.e., vaccination or natural infection). The noninfected group presented a nonsignificantly higher number of participants with a previous COVID disease.

**TABLE 1:** Demographic data of household members included in this study, separated by the 14‐day SARS‐CoV‐2 status.

	Household members (*n* = 20)		
Number of household members [median]	2 [2–3]		
COVID‐19 qPCR test	Positive (*n* = 10)	Negative (*n* = 10)	p
Female (%)	20	30	ns^a^
Number of Vaccinations [median, p25–75]	2.5 [1.75–3]	2 [2–3]	ns^b^
Days from latest vaccination until first test [median, p25–75]	374 [341–475.5]	409 [339–536.5]	ns^b^
Prior COVID‐19 infection	4	7	ns^a^
Days from latest immunogenic event until first test [median, p25–75]	347 [338.5–387]	312 [192.5–425.5]	ns^b^
Number of comorbidities	1 [0–2.25]	0.5 [0–1.25]	ns^b^

Abbreviation: ns, nonsignificant.

^a^ Calculated by Fisher's exact test.

^b^ Calculated by unpaired *t*‐test.

### 2.2 Not‐Infected Household Members Show Multiple Differently Expressed Features of Adaptive Humoral Immunity

**FIGURE 2:**
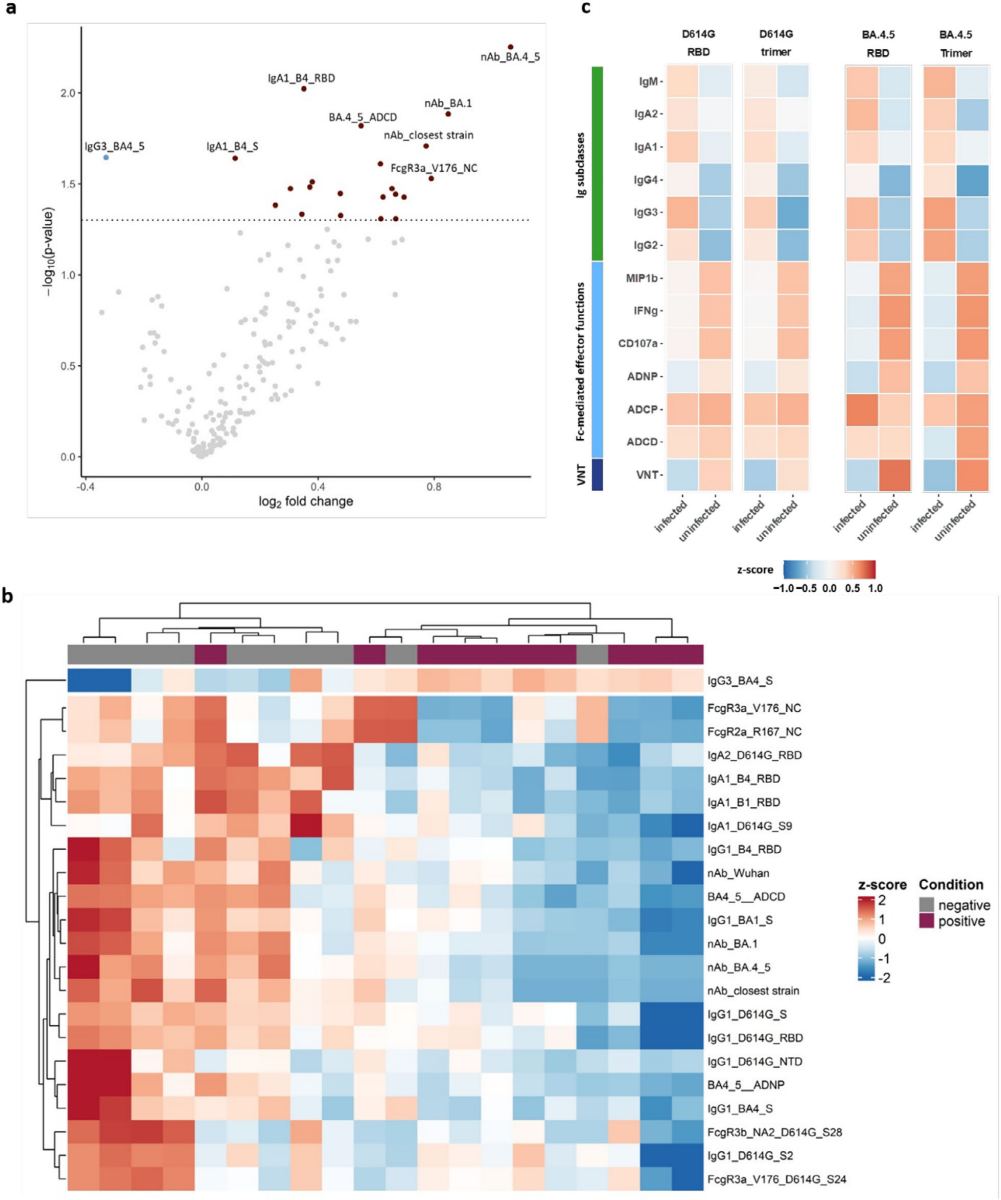
Differentially expressed features in SARS‐CoV‐2‐infected household members at initial sampling. (a) This figure displays a volcano plot of differentially expressed features in household members who were or became SARS‐CoV‐2 positive within 14 days after initial testing. The dotted line marks a *p*‐value threshold of 0.05. Grey dots represent nonsignificant features. Blue dots indicate features significantly upregulated in positive household members, while red dots represent features significantly upregulated in negative household members. (b) A heatmap illustrating the significantly differentially expressed features identified in the volcano plot. Feature levels were log‐transformed. Each column corresponds to a participant, and each row represents feature levels. Column annotations denote the condition of each participant. Clustering was performed based on Euclidean distance. (c) Heatmaps of normalized average IgG1‐corrected values (feature/IgG1) for each feature are shown for spike trimers and RBD of WT (D614G) and Omicron BA.4.5, comparing infected (*n* = 10) to noninfected (*n* = 10) individuals. Data from Luminex bead assays, ADCD, and virus neutralization assays were log10 transformed before IgG1 normalization.

We quantified various features of spike‐specific humoral immunity in the initial serum samples, including a systems serology approach, levels of antibodies targeting different virus epitopes and different SARS‐CoV‐2 variants, and respiratory viruses such as RSV (Table S1). Additionally, nAbs targeting different SARS‐CoV‐2 spike strains were analyzed. Of 198 features, 21 were nominally upregulated in the negative group compared with the positive, and one was downregulated (unadjusted *p* < 0.05; Figure 2a); after correction for multiple testing, none remained significant in the full cohort. Amongst those, seven upregulated features were IgG1 binding titers, targeting different SARS‐CoV‐2 epitopes or strains, and four were related to binding of IgA1 antibodies. Additionally, the nAb concentrations against all tested SARS‐CoV‐2 strains were increased in the not‐infected group. Figure 2b shows the normalized (Z‐score) levels of the nominally differential features in individual sera. Hierarchical clustering indicates clear stratification of positive or negative household members by these features alone. IgG3—which is the first subclass in a naïve immune response—against BA.4/5 spike was higher in infected household members. Conversely, features associated with negative household members were IgG1 binding titers, neutralizing titers, and Fc effector functions (complement deposition: ADCD and neutrophil phagocytosis: ADNP) as well as IgA features against BA4/5 and the ancestral D614G.

IgG1 responses are usually the most dominant among all IgG subclasses. To understand if differences in antibody responses are merely a reflection of overall antibody abundance or mechanistic differences in antibody functions, such as FcgR binding, Fc function, or neutralization, we normalized the functional data to the IgG1 levels specific for D614G trimeric spike or RBD, and for BA.4/5 trimeric spike or RBD. Thereupon, we compared the z‐scores of uninfected and infected groups (Figure 2c). We identified Fc‐mediated effector functions and the virus‐neutralizing titers (VNT) to be more robust in uninfected participants, which is in line with the findings of Figure 2b, and additionally supports the importance of functional antibodies as correlates of protection against transmission.

### 2.3 nAb as Correlate of Protection

**FIGURE 3:**
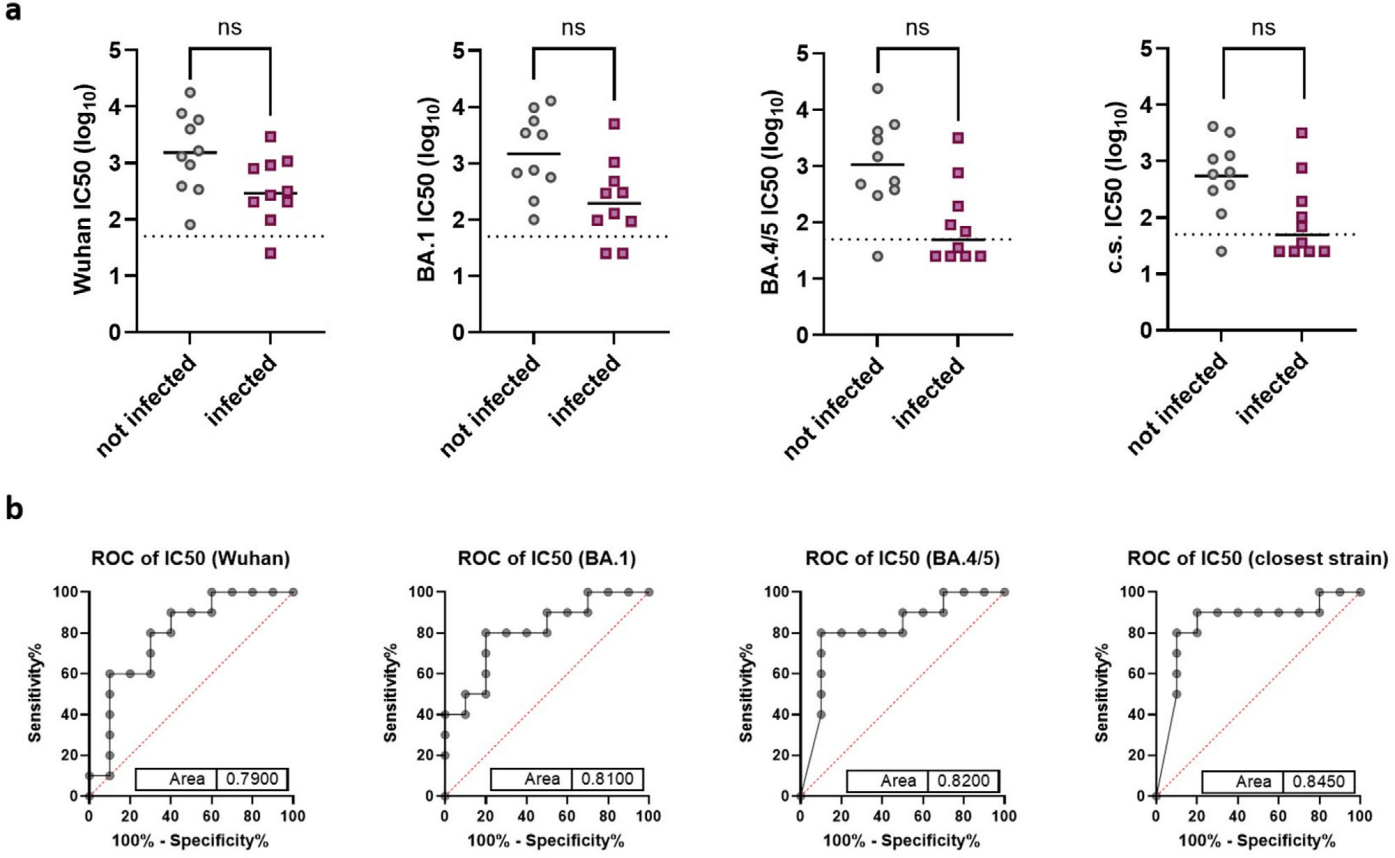
Neutralizing antibody concentrations in household members at initial sampling. (a) Neutralizing antibody (nAb) concentrations were measured using pseudovirus neutralization assays against SARS‐CoV‐2 spike variants Wuhan, Omicron BA.1, and BA.4/5, as well as a relatively closely related strain. Participants were categorized as not infected (grey dots) or infected (purple squares). The dotted line indicates the limit of confidence. For analysis, the data were log‐transformed, and a Mann–Whitney *U*‐test with Benjamini–Hochberg correction was conducted. The black line represents the median for each group. (b) ROC analysis of nAb concentrations, with the area under the curve shown in the plot. *p < 0.05.

To further investigate the importance of nAbs, we individually analyzed their concentrations. All concentrations were elevated in participants who did not get infected (Figure 3a) even against the original Wuhan strain, which by the time of sampling was no longer present. Interestingly, the closer the tested strains got to the strain by which the index person was infected and to the variant BA.4/5 circulating at that time, the more prominent the difference appeared. By looking at their prognostic potential with a receiver operating characteristic (ROC) curve, all measured nAb concentrations discriminated the two groups (AUC 0.79–0.82; Figure 3b), although none of these differences remained significant after correction for multiple testing in the full cohort.

### 2.4 High IgM Values Confound Predictive Potential of Serological Assays

**FIGURE 4:**
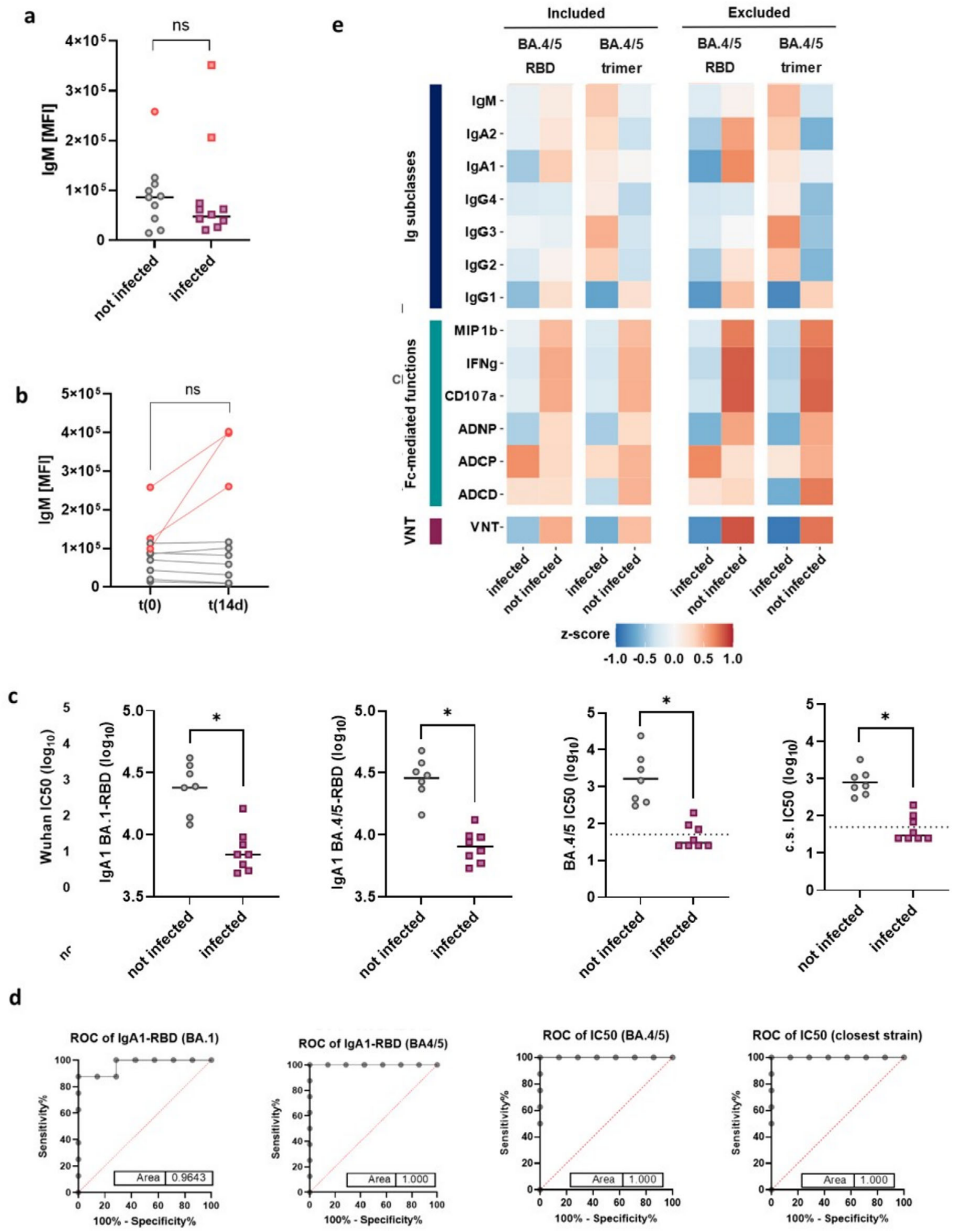
nAb concentration without IgM outliers. (a) IgM levels against the SARS‐CoV‐2 nucleocapsid were measured using Luminex. Participants were categorized as not infected (grey dots) or infected (purple squares). One participant in the not infected group (red dot) and two outliers in the infected group (red squares) presented a high IgM level. A Mann–Whitney *U* test was conducted for analysis. (b) IgM levels of negative household members at the first time point (t(0)) and after 2 weeks (t(14d)). Three participants seroconverted over time and are marked in red. A paired *t*‐test was performed. (c) Participants were categorized as not infected (white dots) or infected (purple squares); participants with high IgM were excluded from the analysis. The dotted line indicates the limit of confidence. For analysis, the data were log‐transformed, and a Mann–Whitney *U* test with Benjamini–Hochberg correction was conducted. The black line represents the median for each group. (d) ROC analysis of nAb concentrations, with the area under the curve shown in the plot. (e) Heatmaps of normalized average IgG1‐corrected values (feature/IgG1) for each feature are shown for spike trimers and RBD of Omicron BA.4.5, comparing normalization including IgM‐high participants (left) to excluding IgM‐high and/or seroconverted participants (right). Data from Luminex bead assays, ADCD, and virus neutralization assays were log10 transformed before IgG1 normalization. MFI = median fluorescent intensity; *p < 0.05; **p < 0.01; ***p < 0.001.

As the majority of our infected participants were already SARS‐CoV‐2 positive at the initial time point, we expected high IgM levels in some of them, limiting our ability to mainly analyze the nAb fraction, which was present before the exposure. Hence, we checked the IgM levels targeting the nucleocapsid of the household members to identify outliers with very high IgM. In Figure 4a, we show that the levels were not significantly different between the two groups. Nevertheless, we identified three outliers (one in the noninfected, two in the infected) that had extremely elevated IgM in contrast to the rest of their respective group. Hence, we performed assays where we used 2‐ME to eliminate IgM from the serum [14]. We validated the results by ELISA (Figure S3a), confirming that IgM was depleted with modest effect on IgG concentrations. Next, all pseudovirus neutralization assays were repeated with 2‐ME‐treated serum (Figure S3b). However, the difference between the two groups did not drastically increase, and in the case of the closest strain even decreased (ROC Wuhan *p* = 0.0284; BA.1 *p* = 0.0233; BA.4/5 *p* = 0.0156; c.s. *p* = 0.0233). Interestingly, individuals with low neutralizing capacity showed increased neutralization upon IgM depletion (Figure S3b).

We considered that relying on PCR values alone might not be sufficient to identify individuals with an asymptomatic infection, especially if they were positive for a brief period between measurements. Hence, we assessed the group of qPCR‐negative members for seroconversion within 14 days, as an indication of an asymptomatic recent disease. We identified three participants who had an increase of IgM within these 2 weeks (Figure 4b). One of them presented an outlier IgM concentration at onset, whereas the other two showed no obviously elevated IgM concentrations at the time of the first sampling. We compared the serology results after excluding the five participants with outlier IgM levels across both groups (leaving *n* = 7 noninfected and 8 infected), as their recent infection might have compromised their serology results. Upon this adjustment, the differences between infected and uninfected groups generally increased, and 24 features remained significant after correction for multiple testing (Table S2), especially Omicron‐RBD‐specific IgA1 and neutralizing titers against Omicron BA.4/5 or the closest strain (Figure 4c). Moreover, the ROC analysis indicated no overlap between groups (AUC = 1.00) in assays with BA4/5 IgA1, BA.4/5 neutralization, or the neutralization against the closest strain (Figure 4d) (*q* = 0.031 after correction). To see how censoring of individuals with recent IgM seroconversion affects other functional assays shown in Figure 2c, we repeated the z‐score comparison of results upon normalization to IgG1 responses to the full‐length BA.4/5 spike or RBD (Figure 4e). Upon exclusion of recent seroconverters, we noticed an obvious increase in differences in VNT, but also in cytokine and degranulation responses of NK cells in coculture with sera (Mip‐1b, IFNγ, CD107), and to a lesser extent of antibody‐dependent neutrophil phagocytosis (ADNP) between infected and uninfected individuals.

## 3 Discussion

SARS‐CoV‐2 vaccination has been suggested to primarily protect against symptomatic or severe disease rather than preventing infection altogether [15]. Consequently, vaccinations are mainly recommended for individuals at high risk for severe COVID‐19, such as those over 60 years old, immunocompromised individuals, or healthcare workers interacting with high‐risk groups [3]. Previous studies with a similar aim, but no exposure documentation, have highlighted the importance of nAbs in disease prevention [16, 17, 18]. This study aimed to determine whether any serological parameters of acquired immunity may predict protection from breakthrough infections. A previous study showed that healthcare workers with breakthrough SARS‐CoV‐2 infections had significantly lower IgG antibody titers, but showed substantial overlaps between groups [19]. However, they did not document the exposure of study participants to the virus, and it remained unclear if participants who remained negative were protected by their adaptive immune response or merely unexposed to the virus. Another study that was conducted in Israel in 2021 tested people exposed to a SARS‐CoV‐2‐positive contact in their household during the Delta wave. This study identified nAb and IgG titers as CoPs against breakthrough infection, with some outliers that were infected even when antibody titers were rather high. However, that study tested only antibodies against the ancestral spike antigen, and not the Delta variants, which were in circulation at the time [20]. We conducted our study with households containing a SARS‐CoV‐2‐positive index person, who acted as the disease transmitter, and assessed serology in a variant‐specific manner, showing that responses to BA.4/5 spike antigen, common in our cohort, outperformed the predictive value of ancestral B.1 or even Omicron BA.1.

Importantly, both groups in our study were similar in terms of vaccination status or time elapsed since the last immunogenic event. This suggests that the time since the last vaccination is a rather crude predictor of protection against breakthrough infections. On the other hand, neutralizing titers against a close variant were a very robust correlate of protection, especially among people with no immunological evidence of a recent infection. We propose that our results argue for two important ideas. Firstly, our data argue that high neutralizing titers may protect against any breakthrough infection, and thus impede transmission. Therefore, a high antibody titer induced by a fresh booster shot might prevent not only disease, but also reduce transmission. Secondly, our data suggest that eliciting high neutralizing titers among caregivers of immunocompromised people could limit the spread of the virus to the vulnerable contacts. Consequently, we propose that regular assessments of neutralizing antibody titers of medical personnel and caregivers working with vulnerable populations might identify those who would benefit from additional booster shots; this proposal remains to be tested prospectively.

Since some participants were already infected at the initial time point, we investigated the impact of IgM on neutralization. We observed that IgM levels were elevated in several study participants, indicating recent exposure to the virus. Excluding the participants with high IgM levels from the analysis revealed a remarkably strong differentiation in IgA1 and nAb concentrations between positive and negative household members. This suggests that individuals with high IgM levels may skew the predictive value of systemic IgA1 and neutralizing Ab as correlates of protection. To explore this further, we also depleted IgM using 2‐ME and repeated the neutralization assay [14]. Overall, no significant differences were observed compared with the IgM‐containing assays. This finding aligns with the results of Klingler et al. [21], who demonstrated that IgM and IgG are both important for virus neutralization. We propose that the participants with high IgM had high neutralization efficacy due to recent infections and that any serological diagnostics for CoP need to include IgM testing to identify people with a recent exposure to the virus.

The role of IgA antibodies for protection from SARS‐CoV‐2 infection is not unambiguous. Despite mounting protective responses, intramuscular mRNA vaccines often induce only modest mucosal IgA responses. Yet, IgA levels in the nose or lungs have been associated with lower risk of infection, and dimeric IgA has been shown to neutralize SARS‐CoV‐2 more potently than monomeric IgA. In our study, we focus on hybrid immunity, in which IgA levels may be strongly influenced by recent infection or exposure in addition to vaccination. Furthermore, systemic IgA is often only weakly associated with mucosal IgA measured in nasal washes, saliva, or bronchoalveolar lavage fluid, which may be explained by local IgA production by tissue‐resident plasma cells and active transport of polymeric IgA across mucosal surfaces. Since we did not analyze mucosal samples, our interpretation is limited to systemic IgA responses and should not be interpreted as a direct surrogate for mucosal IgA. However, our findings support a potential role for systemic IgA as part of the broader humoral immune profile, including neutralizing and nonneutralizing antibody functions and T cells, associated with protection.

Our study has several limitations. We performed exhaustive immunological analyses on samples from a rather small cohort. In consequence, the statistical power was low, and some of the features that were classified as nonsignificant may well differ between people who are protected against household transmission and those who are not. The low statistical power also limits our ability to define quantitative thresholds in antibody titers needed for protection. Moreover, since we used a neutralization assay performed with pseudo‐typed viruses, the standardization of this assay for quantitative purposes may present technical challenges. On the other hand, the IgA1 serology may provide a more actionable biomarker for predictive diagnostics. We did not interview the household member about their behavior, such as masking, distancing, or isolation from the index case, and we did not perform PCR analyses on a daily basis, thus potentially omitting cases that resolved very rapidly. However, since we identified seroconverted individuals, this was probably not a major issue. Additionally, as antibody‐mediated protection is variant‐specific [10, 11, 22], our associations may not transfer directly to other variants. Our study was conducted in late 2022, during the prevalence of the Omicron BA.4/5 variant, before the emergence of currently circulating variants. Hence, a similar study performed on samples from current and upcoming variants is required to solidify our conclusions.

Nevertheless, our findings demonstrate that variant‐specific IgA1 and nAb concentrations strongly correlate with protection against SARS‐CoV‐2 infection. Contrary to current vaccination recommendations, we propose that serology testing for IgA1 and boosting those with low concentrations may prevent not only disease manifestations, but also effectively block transmission. Notably, serological testing with the variant that is currently prevalent provides high accuracy, eliminating the need for an index person‐specific test and simplifying this diagnostic approach.

## 4 Material and Methods

### 4.1 Study Participants and Study Design

This study included household members of individuals who tested positive for SARS‐CoV‐2. Patients with SARS‐CoV‐2‐like symptoms were tested in the Emergency Department of the Clinical Hospital Center Rijeka. Household members (older than 18 years) of index cases who appeared SARS‐CoV‐2 positive in routine screenings were asked to visit the hospital within 48 h. Index (*n* = 18) and household contacts (*n* = 20) were tested via nasopharyngeal swab, by rapid test (Hangzhou AllTest Biotech Co., Ltd) and a fast RT‐qPCR, essentially as described [23], to determine the SARS‐CoV‐2 status. Additionally, a blood sample was obtained. The household members who were SARS‐CoV‐2 negative at the first time‐point (*n* = 12 at 48 h) were asked to come back after 14 days or in between if they experienced symptoms to get tested a second time. A schematic overview of the study design can be seen in Figure 1 and Figure S1. The recruitment of participants occurred in December 2022. Throughout, protection refers to protection of an exposed household contact from acquiring infection following documented exposure to a PCR‐confirmed index case; onward transmission from infected contacts was not measured.

### 4.2 Sample Preparation

Nasal swab samples were either stored at 4°C for immediate use or aliquoted and stored at −80°C. The variant by which the index person was infected was identified by next‐generation sequencing (NGS) using the NEBNext ARTIC SARS‐CoV‐2 FS Library Prep Kit for Illumina with VarSkip 2b primers (New England Biolabs, MA, USA). Paired‐end sequencing was performed on an Illumina NextSeq 1000 (Illumina, CA, USA). For assembly, CoVpipe v3.1.0 (Robert Koch‐Institut, Germany; https://www.gitlab.com/RKIBioinformaticsPipelines/ncov_minipipe) was used with standard parameters, while lineage assignment was done with pangolin v4.3 and pangolin‐data v1.21 [24]. Whole blood samples were incubated for 30–60 min at room temperature. Serum and blood clot were separated by centrifugation at 1500*g* for 10 min. The supernatant was aliquoted and stored at −80°C. The samples were shipped on dry ice to the Helmholtz Center for Infection Research in Braunschweig for further analyses.

### 4.3 Demographic Data and Outcomes

Demographic data of all participants are listed in Table 1. Data were obtained through questionnaires, which were administered at the first and second sampling dates either in person or through follow‐up phone calls.

### 4.4 Neutralization Assay

Neutralization assays were performed according to a previously published protocol [25]. The serum samples were heat‐inactivated at 56°C and 500 rpm for 30 min, diluted in 1:10 DMEM (1% Penicillin‐Streptomycin, 1% L‐Glu, 5% FBS), and stored at 4°C or −80°C for long‐term storage. Sera were twofold‐diluted in a 96‐well plate ranging from 1:100 to 1:102400 and incubated with pseudo‐virus particles (600 pfu ± 30%) for 1 h at 37°C. A control showing no inhibition by incubation in the absence of serum was used. Serum‐virus samples were transferred to a VeroE6 96‐well plate (80% confluent) and further incubated at 37°C for 24 h, after which the plates were fixed with 4% paraformaldehyde (PFA) and kept at 4°C until readout. To count GFP^+^ infected cells, the IncuCyte S3 (Sartorius) was used by performing whole‐well scans (4× magnification) in phase‐contrast and green‐fluorescence settings (300 ms exposure). The IncuCyte GUI software (version 2019B Rev1 and 2021B) performed automated counting of GFP fluorescent foci. The pseudo‐virus neutralization titer 50 (PVNT50) was calculated by a nonlinear regression model. The lower limit of confidence (LLOC) was set at a PVNT50 of 50. Nonresponders were given a PVNT50 of 25 for visualization purposes.

### 4.5 IgM Depletion in Serum Samples

For certain experiments, only the IgG fraction of the nAbs was supposed to be measured. Therefore, IgM was depleted using beta‐mercaptoethanol (2‐ME) on heat‐inactivated serum [14]. Serum was incubated with 2‐ME (final concentration of 140 mM) for 1 h at room temperature and 300 rpm. Afterwards, the serum was diluted 1:10 with DMEM (1% Penicillin‐Streptomycin, 1% L‐Glu, 5% FBS) and immediately used for neutralization assays.

### 4.6 SARS‐CoV‐2 Specific IgM and IgG ELISA

To validate the efficiency of the IgM depletion by 2‐ME, an enzyme‐linked immunosorbent assay (ELISA) was performed. Therefore, serum was treated with 2‐ME as explained before, and the Human SARS‐CoV‐2 Spike (Trimer) IgM ELISA Kit (#BMS2324, Invitrogen, Waltham, United States) and Human SARS‐CoV‐2 Spike (Trimer) IgG ELISA Kit (#BMS2325, Invitrogen, Waltham, United States) were performed as per the manufacturer's instructions. The assay was run on exemplary participants with high, medium, and low IgM based on the Luminex assay, in technical duplicates. The Infinite F50 (Tecan, Männedorf, Switzerland) was used for readout of the ELISA plates.

### 4.7 Detection of Antigen‐Specific Antibody Isotypes and Fc‐Receptor Binding

Human serum samples were profiled for antigen‐specific antibody isotypes and binding of Fcγ receptors with a custom antigen panel as described in a previous study [26]. Recombinant antigens were obtained commercially (Sino Biological, Beijing, China). The custom antigen panel consisted of recombinant SARS‐CoV‐2 trimeric spikes (D614G, B1.1.529 and BA.4/BA.5/BA.5.2), receptor binding domain (RBD) (D614G, B1.1.529 and BA.4/BA.5/BA.5.2), N‐terminal domain (NTD) (2019‐nCoV), nucleocapsid (NC) (2019‐nCoV), S1 (D614G) and S2 domains (2019‐nCoV), the trimeric spike of the human coronaviruses HKU1 (N5) and OC43, and the respiratory syncytial virus (RSV) prefusion protein (preF). For the Luminex assay, 10 µg antigen was covalently coupled to 2 million carboxylated magnetic microspheres (Diasorin, Italy) by carboxy‐coupling as described in a previous study [26].

Serum samples were diluted with assay buffer (PBS, 0.1% BSA) depending on the detector to 1:500 for IgG1, 1:100 for IgG2‐4, IgA1‐2 and IgM, and 1:1000 for the Fcγ receptors. In a 384‐well plate, 5 µL of diluted sample was incubated overnight with a pool of the antigen‐coupled microspheres (1,000 beads per antigen per well) at 4°C. Plates were washed three times with PBS‐T (PBS, 0.05% Tween‐20) and incubated for 1 h with 40 µL of PE‐conjugated secondary detector (1 µg/mL) against IgG1 (RRID: AB_2796628), IgG2 (RRID: AB_2796639), IgG3 (RRID: AB_2796701), IgG4 (RRID: AB_2796693), IgM (RRID: AB_2796577), IgA1 (RRID: AB_2796656), and IgA2 (RRID: AB_2796664) (all Southern Biotech, AL, USA). To detect Fc receptor interaction, streptavidin‐PE:biotin‐coupled FcγRIIa (H167), FcγRIIa (R167), FcγRIIb/c, FcγRIIIa (F176), FcγRIIIa (V176), FcγRIIIb (NA1), and FcγRIIIb (NA1) (all AcroBiosystems, DE, USA) were used (1 µg/mL). Upon incubation, immune complexes were washed three times with PBS‐T using the HydroSpeed automated plate washer (Tecan, Switzerland), resuspended in assay buffer, and acquired at the ID7000 Spectral Cell Analyzer (Sony Biotechnology, CA, USA).

### 4.8 Biotinylation and Bead‐Coupling of Antigens

For antibody‐dependent complement deposition (ADCD), antibody‐dependent THP‐1 cell phagocytosis (ADCP), and ADNP, the spike trimers of wild‐type SARS‐CoV‐2 (D614G) and Omicron variant BA.4/5 were biotinylated using the NHS‐Sulfo‐LC‐LC kit (Thermo Fisher, MA, USA) according to the manufacturer's instructions. After the reaction, excess biotin was removed by size exclusion using Zeba spin desalting columns with a 7 kDa cut‐off (Thermo Fisher). 10 µg of biotinylated antigen was coupled to 10 µL of 1.0 µm NeutrAvidin‐labeled microspheres for 2 h at 37°C.

### 4.9 Antibody‐Dependent Complement Deposition

The ability of antigen‐specific antibodies in the human serum samples to induce the complement cascade was assessed as described in a previous study [27]. Briefly, immune complexes were formed in a 96‐well round‐bottom plate by incubating antigen‐coated microspheres with 10 µL of 1:10 diluted human serum samples for 2 h at 37°C. Reconstituted guinea‐pig complement (Cedarlane, ON, Canada) was cleared by centrifugation, transferred to GVB++ buffer (Novateinbio, MA, USA), and incubated with washed immune complexes for 20 min at 37°C and 5% CO_2_. Complexes were then washed once with 15 mM EDTA in PBS. Deposited C3 on beads was stained with 1:100 diluted anti‐guinea pig C3a‐FITC antibody (MP Biomedicals, CA, USA) and afterwards fixed with 4% paraformaldehyde (PFA). C3 deposition was then determined as mean fluorescent intensity (MFI) on an ID7000 Spectral Cell Analyzer (Sony Biotechnology).

### 4.10 Antibody‐Dependent Neutrophil‐Phagocytosis

Neutrophil‐mediated phagocytosis of serum antibodies was determined as described in a previous study [28]. Antigen‐coated yellow‐green fluorescent microspheres were incubated with 10 µL of 1:50 diluted human serum for 2 h at 37°C and 5% CO_2_. Primary neutrophils were obtained by lysis of whole blood with ammonium–chloride–potassium (ACK) buffer (Biomol, Germany) and transferred to R10 media (RPMI 1640, 10% FCS, 1x GlutaMax, 1x Penicillin‐Streptomycin, 10 mM HEPES). The immune complexes were washed by centrifugation and incubated with 50,000 neutrophils for 1 h at 37°C and 5% CO_2_. Next, neutrophils were stained with 1:100 diluted anti‐human CD66b‐Pacific Blue antibody (BioLegend, CA, USA, RRID: AB_2563294) and fixed with 4% PFA for 20 min. Phagocytic activity of neutrophils was measured on an ID7000 Spectral Cell Analyzer (Sony Biotechnology). A phagocytosis score was calculated as the product of the frequency of bead‐positive neutrophils and the MFI of bead‐positive neutrophils.

### 4.11 Antibody‐Dependent THP‐1 Cell‐Phagocytosis

The level of serum antibody‐induced phagocytic activity of THP‐1 monocytes was detected as previously described [5]. In brief, antigen‐coated yellow‐green fluorescent microspheres were combined with 10 µL of 1:100 diluted human serum in a 96‐well round‐bottom plate and incubated for 2 h at 37°C and 5% CO_2_. Next, 25,000 cultured THP‐1 cells (#ACC 16, DSMZ, Germany) in R10 media were added and incubated overnight at 37°C and 5% CO_2_. THP‐1 cells were then fixed with 4% PFA for 20 min, washed and resuspended in PBS before acquisition on an ID7000 Spectral Cell Analyzer (Sony Biotechnology). A phagocytosis score was calculated for THP‐1 monocytes in the same way as for neutrophils.

### 4.12 Antibody‐Dependent NK Cell‐Activation (ADNKA)

To investigate antibody‐mediated NK cell activation, 96‐well flat‐bottom high‐binding plates were coated with 50 µL (3 µg/mL) of either the spike trimer of wild‐type SARS‐CoV2 (D614G) or Omicron variant BA.4/5. After blocking with 5% BSA‐PBS for 2 h, 50 µL of 1:30 diluted human serum samples were added and incubated overnight at 4°C. Primary NK cells were derived from leukocyte reduction cones of healthy donors using the RosetteSep NK cell enrichment kit (STEMCELL Technologies, MA, USA) according to the manufacturer's instructions, followed by density gradient centrifugation. The enriched cells were then stimulated with 1 ng/mL of recombinant human IL‐15 (BioLegend) overnight at 37°C and 5% CO_2_. After washing the immune complexes three times, 50,000 NK cells were added to each well and incubated with 2 µg Brefeldin A (Sigma‐Aldrich, MA, USA), 1:80 GolgiStop (BD Biosciences, NJ, USA), and 1:11 diluted anti‐human CD107a‐BV605 (BioLegend, RRID: AB_2563851) for 5 h at 37°C and 5% CO_2_. To define NK cells as CD3‐CD56+, the cells were stained for 15 min with 1:40 diluted anti‐human CD3‐APC‐Cy7 (BioLegend, RRID: AB_830755) and 1:20 diluted anti‐human CD56‐PE‐Cy7 (BioLegend, RRID: AB_2857328). After fixation and permeabilization with fixation medium (Invitrogen) and permeabilization medium (Invitrogen), respectively, NK cells were stained intracellularly with 1:200 anti‐human MIP‐1β‐BV421 (BD Biosciences, RRID: AB_2737877) and 1:50 anti‐human IFN‐γ‐PE (BioLegend, RRID: AB_315440) for 15 min. Finally, samples were washed, resuspended in PBS, and the MFI of extracellular and intracellular markers was acquired on an ID7000 Spectral Cell Analyzer (Sony Biotechnology).

### 4.13 Statistical Analysis

All data were log10‐transformed. For group comparisons, two‐sided Mann–Whitney *U* tests were used, and *p*‐values were adjusted across the 198 features by the Benjamini–Hochberg procedure (*q* < 0.05 considered significant). Given the number of features relative to participants, no multivariable model was fitted. Data representation and statistical analysis were done using R software v4.3.2 or GraphPad Prism v10.2.2 (GraphPad Software, CA, USA).

### 4.14 Ethical Considerations

The Institutional Review Board of the Rijeka Clinical Hospital Center (2170‐29‐02/1‐22‐2) as well as the Hanover Medical School (10783_BO_K_2023) approved this study. Written informed consent was obtained from all participants.

## Author Contributions

**Henrike Maaß, Mate Lerga, Manuela Harries, Berit Lange, Martina Pavletic, Luka Cicin‐Sain**: conceptualization. **Henrike Maaß, Imke Hinrichs, Mate Lerga, Tatjana Prinke, Najat Bdeir, Richard Egelkamp**: investigation. **Henrike Maaß, Imke Hinrichs, Luka Cicin‐Sain**: formal analysis. **Mate Lerga, Martina Pavletic**: resources. **Henrike Maaß, Luka Cicin‐Sain**: data curation. **Henrike Maaß**: Writing – original draft preparation. **Luka Cicin‐Sain**: Writing – review and editing. **Mate Lerga, Luka Cicin‐Sain**: supervision. **Yannic C. Bartsch, Luka Cicin‐Sain**: funding acquisition. All authors have read and agreed to the published version of the manuscript.

## Conflicts of Interest

Berit Lange is a member of the Expert Council “Health and Resilience”, Federal Chancellery, as well as a member of the Standing Vaccination Commission at the Robert Koch Institute. Berit Lange is the elected (Deputy) President of the German Society for Epidemiology (DGEpi), deputy in 2023 and 2026, president in 2024 and 2025, and part of the pool of experts of the Federal Ministry of Education and Research (BMBF) for consultations on pandemic preparedness and the overall responsiveness of health research to health crises. Berit Lange is also a member of the Advisory Board for the Pact for Public Health (Pakt ÖGD), Federal Ministry of Health (BMG), an elected Speaker of the Modelling Network for Severe Infectious Diseases in Germany (MONID), a member of the DZIF Internal Advisory Board, and an elected member of the steering committee of TBNet. The remaining authors declare no conflicts of interest.

## Supporting information




**Supporting File 1**: eji70269‐sup‐0001‐SuppMat.docx.

**Supporting File 2**: eji70269‐sup‐0002‐Table S2.xlsx.

## Data Availability

The dataset used is available in the supplementary file and from the corresponding author on reasonable request.
